# Co-morbidity associated with development of severe COVID-19 before vaccine availability: a retrospective cohort study in the first pandemic year among the middle-aged and elderly in Jönköping county, Sweden

**DOI:** 10.1186/s12879-023-08115-0

**Published:** 2023-03-14

**Authors:** Dennis Nordvall, Dan Drobin, Toomas Timpka, Robert G. Hahn

**Affiliations:** 1 Qulturum Development Department, Region Jönköping County, Jönköping, Sweden; 2 Region Jönköping County, Division of Surgical Care, Jönköping, Sweden; 3 Department of Medical and Health Sciences, Linköping University, Linköping, Sweden; 4 Research Unit, Södertälje Hospital, Karolinska Institutet at Danderyds Hospital (KIDS), 152 86 Södertälje, Sweden

**Keywords:** Covid-19, lipids, Diabetes type 2, Epidemiology

## Abstract

**Background:**

In preparation of future pandemics, it is important to recognise population-level determinants associated with development of severe illness before efficient vaccines and evidence-based therapeutic measures are available. The aim of this study was to identify pre-pandemic diagnoses recorded in a middle-aged and elderly population that were associated with development of severe COVID-19 during the first pandemic year.

**Methods:**

A cohort study design was used. Severe COVID-19 was defined as a course of illness that resulted in hospital admission or death. A retrospective analysis was performed that comprised all individuals aged 39 years and older (N = 189,951) living in Jönköping County, Sweden. All diagnosed morbidity recorded in contacts with health care during the pre-pandemic year 2019 was used to identify which diagnoses that were associated with development of severe COVID-19 in the first pandemic year 2020. The analyses were performed separately for each diagnosis using binary logistic regression with adjustment for sex and age.

**Results:**

Severe COVID-19 was suffered by 0.67% (N = 1,280) of the middle-aged and elderly population in the first pandemic year. Individuals previously diagnosed with dementia, cerebral palsy, kidney failure, type 2 diabetes mellitus, hypertension, and obesity were at higher risk of developing severe COVID-19. For patients with Type 2 diabetes mellitus, the odds ratio (OR) was 2.18 (95% confidence interval, 1.92–2.48). Type 1 diabetes mellitus was not associated with increased risk.

**Conclusion:**

Diagnoses suggesting service provision at long-term healthcare facilities and co-morbidity with components of the metabolic syndrome were associated with an increased risk of developing severe COVID-19 in a middle-aged and elderly population before vaccines were available.

**Supplementary Information:**

The online version contains supplementary material available at 10.1186/s12879-023-08115-0.

## Background

The COVID-19 pandemic has profoundly influenced social life, economy and health services worldwide. Although individuals infected with SARS-CoV-2 may suffer only mild illness, many develop severe COVID-19 requiring hospital admission and possibly leading to death. The course of severe illness is characterized by fever, cough, and dyspnoea followed by dysregulation of the immunological response (“cytokine storm”) causing sudden deterioration [1]. Much effort has been made to limit the spread of the SARS-CoV-2 virus through vaccination programs [2] and to improve the clinical treatment of COVID-19 with, e.g., provision of nirmatrelvir-ritonavir [3] during the early course of illness and corticosteroids in the later phases [4]. However, in preparation for next pandemic, it is important to identify determinants that were associated with development of severe illness before efficient preventive and therapeutic measures are available.

Male sex and advanced age have in previous research been found to be associated with increased risk of developing severe COVID-19 in the early pandemic stages [5, 6]. Many studies have also focused on selected diagnoses and their association with fatal outcomes [7, 8] or clinical mortality risk scores for in-hospital patients created based on vital signs, blood chemistry, and limited sets of health conditions [9–11]. In consequence, heterogeneity of the early population-based studies has made it difficult to identify meaningful associations between development of severe COVID-19 and pre-existing morbidity during the initial pandemic phases [5].

The aim of this study was to identify pre-pandemic diagnoses among the middle-aged and elderly in a representative Swedish county associated with developing severe COVID-19 during the first pandemic year. We considered all clinical diagnoses as potential determinants of developing severe COVID-19. The results are to be used to inform planning non-pharmaceutical interventions and the initial stages of vaccination programs during future pandemics.

## Materials and methods

A cohort study design was used for the analysis. The study cohort consisted of all individuals aged 39 years and older residing in Jönköping County, Sweden on December 31st 2019 (N = 189,951). The primary endpoint measure was incident cases of severe covid-19 during 2020.

Severe covid-19 was defined as having been diagnosed with COVID-19 confirmed by Reverse Transcription-Polymerase Chain Reaction (RT-PCR) testing (ICD-10 U07.1) and been admitted to hospital or died.

### Data collection

Demographic data (age, sex) for formal Jönköping County residents aged 39 years and older by December 31st 2019 (n = 189,237) were collected from Statistics Sweden (SCB). Data on all diagnoses recorded in 2019 and on hospital admissions and deaths due to covid-19 in 2020 were collected from the Primary Health Care Registry (44 primary care units) and the Specialised Care Registry (three hospitals) in Region Jönköping Län, the health care provider in Jönköping County. The only missed caregivers were a small number of practicing private physicians.

When comparing the demographic and healthcare records, individuals not included in the formal demographic records were found to have been provided health care in Region Jönköping Län during 2019. These individuals were assumed to remain residing in the county. This resulted in a total study cohort (N = 189,951) that was 0.38% (n = 714) larger than the formally defined demographic number.

The ICD-10-SE diagnoses recorded in the study cohort during 2019 comprised 2,028 different diagnosis codes. The 330 most frequently recorded codes accounted for 90% of all registered diagnoses in the study county during 2019 (Supplement Table S1). Binary variables were created for each of these diagnosis codes at the ICD-10-SE Category level (i.e., three characters). When necessary, the Diagnosis level, i.e. four characters, was also used. These 330 variables were the only representations of diagnosis codes used in the study.

Data on severe COVID-19 were gathered from individuals having in 2020 been admitted to hospital and/or died diagnosed with COVID-19 confirmed by Reverse Transcription-Polymerase Chain Reaction (RT-PCR) test (ICD-10 U07.1).

### Data analyses

The study population was first disaggregated according to sex, age, morbidity in 2019, and severe COVID-19 in 2020 and descriptive statistics presented. Binary logistic regression models were then applied to identify determinants of the primary outcome severe COVID-19 (1, yes; 0, no). The determinant variables included in the models were sex, age, and medical diagnoses recorded in 2019. Models were first produced using age (centiles from 39 years of age; reference category 39–48 years) and sex (1, male; 0, female), respectively, as determinant variables and severe COVID-19 in 2020 as outcome variable. The model using sex as determinant variable was adjusted for age and vice versa. Thereafter separate adjusted models were produced for each and every of the 330 selected diagnoses recorded in 2019 (1, present; 0, not present). These models were adjusted for sex and age and used severe COVID-19 in 2020 (1, present; 0, not present) as outcome variable.

The threshold for significance was set at *P* < 0.0001. The results are reported as the odds ratio (OR) and the 95% confidence interval (95% CI). The statistical analyses were performed using the R software package version 4.0 [12].

## Results

The average age of the middle-aged and elderly study cohort was 61.1 years; the proportion of women was 51%. Eigthy-six percent  (n = 162,967) of the population had a diagnosis recorded in the healthcare information system during the pre-pandemic year. Of these, 6,715 (4.1%) were diagnosed with covid-19 and 1,218 (0.74%) developed severe covid-19 in the first pandemic year. Additionally, 62 (0.2%) of the 26,984 individuals with no recorded contact with the healthcare system in 2019 developed severe COVID-19. In total, 1,280 individuals (0.67%) in the study cohort developed severe COVID-19 during 2020. The fatality proportion was 32% (n = 405).

**Fig. 1:**
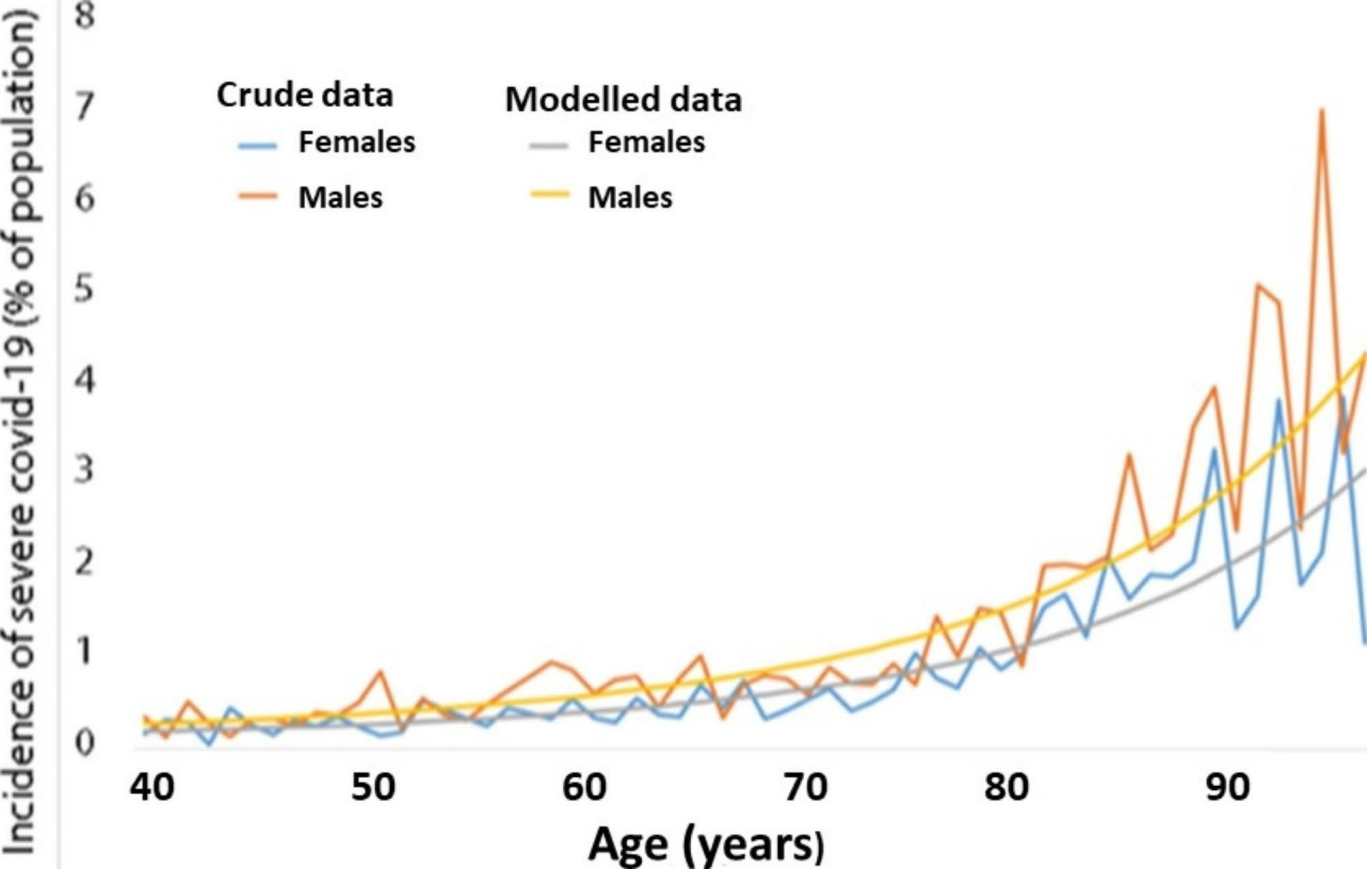
Incidence of severe COVID-19 in the study cohort. Data for individuals ages 39 and older are shown. The data for men and women are separated

Overall, more men than women in the study cohort were admitted to hospital or died, with an age-adjusted OR of 1.39 (95% CI, 1.24–1.55) (Table 1; Fig. 1). Individuals aged 80 years or older had a 7 to 13-fold higher risk of developing severe covid-19 compared with those aged 39–48 years (Table 1).

**Table 1:** Binary logistic regression models of severe COVID-19 displayed by age and sex. The model of sex was adjusted for age and vice versa. Risk estimates were based on 189,951 individuals; 1,280 of these developed severe COVID-19 (0.67%)

Sex / Age group	Odds ratio (95% confidence interval)
Sex	1.39 (1.24–1.55)
Age group	
39–48 years	1
49–58 years	1.53 (1.21– 1.93)
59–68 years	2.09 (1.67–2.63)
69–78 years	2.83 (2.28–3.54)
79–88 years	6.95 (5.64–8.64)
89–98 years	11.93 (9.37–15.25)
99–108 years	12.85 (4.98–27.18)

Male sex and age category 39–48 years are used as reference.

Diagnoses associated with increased risk of severe covid-19 in the age- and sex-adjusted models are listed in Table 2; these included dementia (various forms), kidney failure, cerebral palsy, history of self-harm, type 2 diabetes mellitus, obesity, atherosclerosis, and heart failure.

**Table 2:** Diagnosis codes recorded in 2019 that were associated with severe COVID-19 in 2020 in binary logistic regression models adjusted for age and sex

ICD10 code	Diagnosis	N	OR for severe covid-19 (N = 1,280)	P-value
Diseases of the circulatory system				
I10	Essential hypertension	59,996	1.48 (1.31–1.67)	3 × 10^− 10^
I50	Heart failure	8833	2.08 (1.77–2.44)	2 × 10^− 16^
I70	Atherosclerosis	924	2.63 (1.80–3.72)	2 × 10^− 7^
Diseases of the endocrine system and metabolism				
E11	Diabetes mellitus type 2	18,897	2.18 (1.92– 2.48)	2 × 10^− 16^
E66	Obesity	7494	2.25 (1.83–2.73)	4 × 10^− 15^
E63	Nutritional deficiencies	4089	2.12 (1.70– 2.62)	1 × 10^− 11^
Mental disorders				
F41	Anxiety	13,436	1.54 (1.29–1.82)	2 × 10^− 6^
F03	Unspecified dementia	2229	2.11 (1.64–2.67)	2 × 10^− 9^
F01	Vascular dementia	1133	2.69 (1.97– 3.58)	7 × 10^− 11^
Diseases of the respiratory system				
J45	Asthma	10,655	1.52 (1.24–1.85)	3 × 10^− 5^
J44	Chronic obstructive pulmonary disease	6484	1.91 (1.57– 2.30)	4 × 10^− 11^
Digestive diseases				
K59	Obstipation	9504	2.16 (1.84– 2.53)	2 × 10^− 16^
Diseases of the genitourinary system				
N39	Urinary tract infection/incontinence	9374	1.94 (1.64–2.28)	3 × 10^− 15^
N30	Cystitis	8672	1.83 (1.52–2.20)	1 × 10^− 10^
N19	Unspecified kidney failure	3097	1.90 (1.51– 2.37)	2 × 10^− 8^
N18	Chronic kidney failure	2151	2.44 (1.88–3.13)	8 × 10^− 12^
Diseases of the nervous system				
G47	Sleep disorders	9033	1.68 (1.38– 2.03)	8 × 10^− 8^
G30	Alzheimer’s disease	1197	2.66 (1.94– 3.56)	3 × 10^− 10^
G80	Cerebral palsy	165	8.39 (3.53– 16.81)	6 × 10^− 8^
Factors influencing health status				
Z921	Long-term use of anticoagulants	8147	1.77 (1.49– 2.10)	4 × 10^− 11^
Z867	History of pulmonary embolism	3551	1.86 (1.45–2.35)	6 × 10^− 7^
Z99	Dependence on enabling machines	2227	2.23 (1.57– 3.07)	3 × 10^− 6^
Z915	Personal history of self-harm	1263	3.48 (2.09– 5.41)	3 × 10^− 7^
Z94	Transplanted organ	587	4.22 (2.45–6.74)	3 × 10^− 8^
Diseases of the blood and immune mechanism				
D64	Anemia	4750	1.90 (1.54– 2.31)	8 × 10^− 10^
Diseases of the musculoskeletal system				
M10	Gout	3847	2.10 (1.67–2.60)	7 × 10^− 11^

Secondary conditions and symptoms were also found to be associated with increased risk of severe COVID-19, e.g. dependence on enabling technologies and obstipation.

## Discussion

We found that 0.67% of the middle-aged and elderly population in a Swedish county developed severe COVID-19 in the first pandemic year. Old age, male sex, conditions associated with daily care support needs, and diagnoses included in the metabolic syndrome were associated with developing severe COVID-19 before the vaccination program was initiated. The increased risk of severe COVID-19 in individuals with dementia, cerebral palsy, and a history of self-harm may be explained by the high prevalence of long-term care facility residents in these groups. Swedish nursery homes suffered substantial problems with widespread dissemination of SARS-CoV-2 during 2020 [13]. In contrast, the diagnoses associated with the metabolic syndrome, comprising type 2 diabetes mellitus, obesity, and hypertension, indicated an individual frailty for developing a severe course of illness following SARS-CoV-2 infection [14–16]. This observation suggests that in the period before vaccines were available, co-morbidity with disorders having lifestyle-related etiologies were associated with development of severe covid-19. However, this hypothesis requires confirmation in studies accounting for causal mechanisms in the analysis models.

### Determinants of COVID-19 in unvaccinated populations

In a meta-analysis of 76 population-based studies performed before vaccine availability, Booth et al. found increased risk estimates for male sex, advanced age, severe obesity, and active cancer [5]. Some diagnoses we observed to be associated with increased risk (such as diabetes type 2, hypertension, and kidney disease) may not have reached statistical significance in the meta-analysis due to the heterogeneity of the studies included. In comparison, the OpenSAFELY study based on electronic medical record data from 40% of all patients in England reported findings similar to our observations, i.e. that advanced age, male sex, asthma, hypertension, diabetes, recent cancer diagnosis, and reduced kidney function were associated with development of severe COVID-19 [6]. However, it is noteworthy that cancer was not included among the determinants identified in our study. One explanation is that we, due to interpretation issues associated with “Table 2 Fallacy” [17], did not adjust for co-morbidity in our analyses. Other explanations include that the non-pharmaceutical infectious disease control interventions implemented among the cancer patients were successful in the study county, and that the cancer diagnoses was recorded during the pre-pandemic year and many patients with the diagnosis had completed their cancer treatments during the first pandemic year.

### Study strengths and limitations

An important strength of our study is that the data on pre-existing morbidity were collected before the outbreak of the pandemic, which prevents diagnoses caused by COVID-19 to be included in the results. Moreover, the data were derived from both specialised (hospital) care and primary care settings. A large majority of the middle-aged and elderly study population (86%) had data on diagnoses recorded the healthcare system during the pre-pandemic year. We have not been able to identify studies that use pre pandemic diagnosis data from a total middle-aged and elderly population for analyses of determinants of hospitalisation and death due to COVID-19 in the first pandemic year. Regarding limitations, there can have been diseases that remained undiagnosed in the population. Our data are probably more complete for severe diseases than for the less severe diseases. Moreover, morbidity that is diagnosed according to well-defined criteria may also be more complete. For example, physicians may not have registered all diagnoses or been reluctant to record behavioural problems or lifestyle issues, e.g. substance abuse. Unobserved confounding may also have been present. We did not adjust the models with the separate diagnoses as determinants for severe COVID-19 with regards to concurrent co-morbidity or socioeconomic factors, e.g., country of birth, education, or income. Associations between socioeconomic factors and propensity of need for intensive care COVID-19 treatment during the early pandemic has been reported from Sweden [18]. The influence from comorbidity and socioeconomic factors on COVID-19 morbidity during the early pandemic phase before vaccines were available warrant further research.

An important limitation of our study design based on data retrieved from electronic medical records. Criticism has been raised against the OpenSafely study that used a study design similar to ours [6, 17, 19]. The authors collected data on patients who had deceased following that a validated or suspected SARS-CoV-2 infection was recorded in their electronic medical record. Hence, PCR testing had not been uniformly applied to validate the presence of COVID-19 infection. In our study, the endpoint measure included having a positive SARS-CoV-2 test recorded in the electronic medical record. Nonetheless, it was not ascertained whether the infection was the cause of hospitalisation or death. Also, the generalization of our results to other setting must be made with caution, although we believe that the strengths of the study (completeness of data collected from a total population) provide satisfactory external validity. We thus infer that our results are representative of comparable counties.

## Conclusion

Electronic medical record data from primary and specialised care in Region Jönköping County, Sweden, showed that diagnoses suggesting service provision at long-term healthcare facilities and co-morbidity with components of the metabolic syndrome were associated with an increased risk of developing severe COVID-19 among the middle-aged and elderly in the period before vaccines were available. Our results can be used to inform planning of non-pharmaceutical interventions during the early stages of future pandemics caused by pathogens similar to SARS-CoV-2.

## Electronic supplementary material

Below is the link to the electronic supplementary material.

Supplementary Material 1

## Data Availability

The data that support the findings of this study are available from a Jönköping County. Restrictions apply to the availability of these data, which were used under license for the current study, and so are not publicly available. However, data with personal identifiers removed are available from the first author (Dennis Nordvall, who is available at E-mail: dennis.nordvall@rjl.se) upon reasonable request and with permission of Jönköping County.
