# Supplementary material for: Co-morbidity associated with development of severe COVID-19 before vaccine availability: a retrospective cohort study in the first pandemic year among the middle-aged and elderly in Jönköping county, Sweden
Source: BMC Infect Dis. 2023 Mar 14;23:156. doi: 10.1186/s12879-023-08115-0 (PMC10012282; doi:10.1186/s12879-023-08115-0)
Supplement: Supplementary file 1 — Supplementary Material 1 [file 12879_2023_8115_MOESM1_ESM.docx]

| **ICD10 code** | **n** | **Diagnosis** |
| --- | --- | --- |
| I10 | 59996 | Essential hypertension |
| E78 | 27588 | Disorders of lipoprotein metabolism and other lipidemias |
| M79 | 22826 | Oth and unsp soft tissue disorders |
| M54 | 20373 | Dorsalgia |
| E11 | 18897 | Diabetes mellitus type 2 |
| R52 | 15424 | Pain |
| F51 | 14873 | Sleep disorders not due to a substance or known physiological condition |
| F32 | 13823 | Major depressive disorder |
| F41 | 13436 | Anxiety |
| M25 | 13113 | Other joint disorder |
| I25 | 12632 | Chronic ischemic heart disease |
| Z12 | 12241 | Encounter for screening for malignant neoplasms |
| H91 | 11849 | Other and unspecified hearing loss |
| I48 | 11477 | Atrial fibrillation and flutter |
| J45 | 10655 | Asthma |
| Z09 | 10430 | Encntr for f/u exam aft trtmt for cond oth than malig neoplm |
| M17 | 9685 | Osteoarthritis of knee |
| R23 | 9572 | Other skin changes |
| K59 | 9504 | Obstipation |
| R10 | 9474 | Abdominal and pelvic pain |
| N39 | 9374 | Urinary tract infection/incontinence |
| R05 | 9289 | Cough |
| E03 | 9195 | Other hypothyroidism |
| G47 | 9033 | Sleep disorders |
| Z92 | 8917 | Personal history of medical treatment |
| I50 | 8833 | Heart failure |
| N30 | 8672 | Cystitis |
| F43 | 8616 | Reaction to severe stress |
| Z00 | 8476 | Encntr for general exam w/o complaint |
| Z96 | 8431 | Presence of other functional implants |
| H61 | 8365 | Other disorders of external ear |
| Z921 | 8147 | Long-term use of anticoagulants |
| K30 | 8126 | Functional dyspepsia |
| Z46 | 8053 | Encounter for fitting and adjustment of other devices |
| H25 | 8045 | Age-related cataract |
| J06 | 7770 | Acute upper resp infections of multiple and unsp sites |
| R53 | 7628 | Malaise and fatigue |
| R06 | 7610 | Abnormalities of breathing |
| E66 | 7494 | Obesity |
| Z85 | 7489 | Personal history of malignant neoplasm |
| K21 | 7264 | Gastro-esophageal reflux disease |
| Z03 | 7116 | Encntr for medical obs for susp diseases and cond ruled out |
| L30 | 6826 | Other and unspecified dermatitis |
| R07 | 6658 | Pain in throat and chest |
| J44 | 6484 | Chronic obstructive pulmonary disease |
| Z86 | 6308 | Personal history of certain other diseases |
| R42 | 6144 | Dizziness and giddiness |
| H35 | 6053 | Other retinal disorders |
| N40 | 5932 | Enlarged prostate |
| M75 | 5872 | Shoulder lesions |
| Z51 | 5605 | Encounter for other aftercare |
| M19 | 5484 | Other and unspecified osteoarthritis |
| Z95 | 5482 | Presence of cardiac and vascular implants and grafts |
| N95 | 5460 | Menopausal and other perimenopausal disorders |
| T00 | 5066 | Superficial injuries involving multiple body regions |
| M16 | 4973 | Osteoarthritis of hip |
| M81 | 4889 | Osteoporosis without current pathological fracture |
| D64 | 4750 | Anemia |
| H36 | 4701 | Retinal disorders in diseases classified elsewhere |
| J30 | 4555 | Vasomotor and allergic rhinitis |
| T01 | 4472 | Open wounds involving multiple body regions |
| R03 | 4169 | Abnormal blood-pressure reading |
| J18 | 4135 | Pneumonia |
| R00 | 4112 | Abnormalities of heart beat |
| Z72 | 4112 | Problems related to lifestyle |
| E63 | 4089 | Nutritional deficiencies |
| M10 | 3847 | Gout |
| I69 | 3800 | Sequelae of cerebrovascular disease |
| R39 | 3781 | Oth and unsp symptoms and signs involving the GU sys |
| R51 | 3705 | Headache |
| R60 | 3686 | Edema |
| H10 | 3673 | Conjunctivitis |
| L57 | 3655 | Skin changes due to chronic expsr to nonionizing radiation |
| L08 | 3576 | Other local infections of skin and subcutaneous tissue |
| Z867 | 3551 | History of pulmonary embolism |
| W01 | 3485 | Fall on same level from slipping |
| D51 | 3436 | Vitamin B12 deficiency anemia |
| Z91 | 3334 | Personal history of risk-factors, not elsewhere classified |
| D50 | 3291 | Iron deficiency anemia |
| I20 | 3241 | Angina pectoris |
| C61 | 3219 | Malignant neoplasm of prostate |
| H60 | 3139 | Otitis externa |
| M35 | 3115 | Other systemic involvement of connective tissue |
| N19 | 3097 | Unspecified kidney failure |
| M80 | 3088 | Osteoporosis with current pathological fracture |
| H04 | 3058 | Disorders of lacrimal system |
| G43 | 3009 | Migraine |
| B34 | 2996 | Viral infection of unspecified site |
| Z08 | 2921 | Encntr for follow-up exam after trtmt for malignant neoplasm |
| C44 | 2908 | Other and unspecified malignant neoplasm of skin |
| Z45 | 2850 | Encounter for adjustment and management of implanted device |
| L40 | 2831 | Psoriasis |
| M85 | 2758 | Other disorders of bone density and structure |
| R22 | 2568 | Localized swelling |
| Z54 | 2562 | Convalescence |
| I35 | 2558 | Nonrheumatic aortic valve disorders |
| R73 | 2542 | Elevated blood glucose level |
| R41 | 2472 | Oth symptoms and signs w cognitive functions and awareness |
| K57 | 2454 | Diverticular disease of intestine |
| K44 | 2398 | Diaphragmatic hernia |
| W19 | 2386 | Unspecified fall |
| R79 | 2380 | Other abnormal findings of blood chemistry |
| I49 | 2356 | Other cardiac arrhythmias |
| L29 | 2287 | Pruritus |
| F03 | 2229 | Unspecified dementia |
| Z99 | 2227 | Dependence on enabling machines |
| N18 | 2151 | Chronic kidney failure |
| R11 | 2142 | Nausea and vomiting |
| J22 | 2122 | Unspecified acute lower respiratory infection |
| H81 | 2115 | Disorders of vestibular function |
| E55 | 2067 | Vitamin D deficiency |
| R74 | 2043 | Abnormal serum enzyme levels |
| G56 | 2033 | Mononeuropathies of upper limb |
| I83 | 2020 | Varicose veins of lower extremities |
| D52 | 2010 | Folate deficiency anemia |
| R50 | 2004 | Fever of other and unknown origin |
| R20 | 1976 | Disturbances of skin sensation |
| I87 | 1940 | Other disorders of veins |
| R19 | 1939 | Oth symptoms and signs involving the dgstv sys and abdomen |
| H26 | 1931 | Other cataract |
| L20 | 1931 | Atopic dermatitis |
| E10 | 1888 | Diabetes mellitus type 1 |
| G40 | 1847 | Epilepsy and recurrent seizures |
| G62 | 1817 | Other and unspecified polyneuropathies |
| F10 | 1804 | Alcohol related disorders |
| R29 | 1801 | Oth symptoms and signs involving the nervous and ms systems |
| G25 | 1795 | Other extrapyramidal and movement disorders |
| M51 | 1793 | Other intervertebral disc displacement |
| Z866 | 1779 | Personal history of dis of the nervous sys and sense organs |
| E87 | 1743 | Other disorders of fluid |
| Z53 | 1740 | Persons encntr hlth serv for spec proc & trtmt |
| M05 | 1681 | Rheumatoid arthritis with rheumatoid factor |
| Z04 | 1671 | Encounter for examination and observation for other reasons |
| G44 | 1609 | Other headache syndromes |
| H93 | 1599 | Other disorders of ear |
| R13 | 1585 | Aphagia and dysphagia |
| Z48 | 1579 | Encounter for other postprocedural aftercare |
| M72 | 1556 | Fibroblastic disorders |
| R31 | 1547 | Hematuria |
| E53 | 1545 | Deficiency of other B group vitamins |
| A46 | 1511 | Erysipelas |
| K64 | 1507 | Hemorrhoids and perianal venous thrombosis |
| H53 | 1477 | Visual disturbances |
| K29 | 1459 | Gastritis and duodenitis |
| K58 | 1441 | Irritable bowel syndrome |
| R55 | 1432 | Syncope and collapse |
| K40 | 1366 | Inguinal hernia |
| I73 | 1363 | Other peripheral vascular diseases |
| M20 | 1361 | Acquired deformities of fingers and toes |
| I63 | 1358 | Cerebral infarction |
| N20 | 1302 | Calculus of kidney and ureter |
| I95 | 1287 | Hypotension |
| K63 | 1276 | Other diseases of intestine |
| I80 | 1267 | Phlebitis and thrombophlebitis |
| Z915 | 1263 | Personal history of self-harm |
| L21 | 1254 | Seborrheic dermatitis |
| J20 | 1246 | Acute bronchitis |
| K51 | 1218 | Ulcerative colitis |
| R32 | 1215 | Unspecified urinary incontinence |
| I44 | 1213 | Atrioventricular and left bundle-branch block |
| F17 | 1206 | Nicotine dependence |
| R25 | 1205 | Abnormal involuntary movements |
| G30 | 1197 | Alzheimer's disease |
| R30 | 1180 | Pain associated with micturition |
| F01 | 1133 | Vascular dementia |
| R63 | 1130 | Symptoms and signs concerning food and fluid intake |
| H01 | 1128 | Other inflammation of eyelid |
| L71 | 1110 | Rosacea |
| F31 | 1077 | Bipolar disorder |
| K80 | 1076 | Cholelithiasis |
| I34 | 1065 | Nonrheumatic mitral valve disorders |
| H54 | 1061 | Blindness and low vision |
| H02 | 1060 | Other disorders of eyelid |
| L02 | 1060 | Cutaneous abscess |
| L97 | 1057 | Non-pressure chronic ulcer of lower limb |
| J15 | 1031 | Bacterial pneumonia |
| L60 | 1028 | Nail disorders |
| J03 | 1020 | Acute tonsillitis |
| H18 | 996 | Other disorders of cornea |
| Z98 | 993 | Other postprocedural states |
| G45 | 984 | Transient cerebral ischemic attacks and related syndromes |
| L72 | 974 | Follicular cysts of skin and subcutaneous tissue |
| I21 | 973 | STEMI & NSTEMI mocard infrc |
| R45 | 966 | Symptoms and signs involving emotional state |
| I71 | 961 | Aortic aneurysm and dissection |
| M23 | 958 | Internal derangement of knee |
| C50 | 932 | Malignant neoplasm of breast |
| I70 | 924 | Atherosclerosis |
| G55 | 908 | Nerve root and plexus compressions in diseases classd elswhr |
| F900B | 907 | Attention Deficit and Hyperactivity Disorder (ADHD) |
| H66 | 902 | Suppurative and unspecified otitis media |
| Y57 | 871 | Other medicines or drugs |
| I47 | 860 | Paroxysmal tachycardia |
| Z864 | 858 | Personal history of psychoactive substance abuse |
| R49 | 856 | Voice and resonance disorders |
| M13 | 846 | Other arthritis |
| H34 | 829 | Retinal vascular occlusions |
| N10 | 814 | Acute tubulo-interstitial nephritis |
| H65 | 813 | Nonsuppurative otitis media |
| K50 | 806 | Crohn's disease [regional enteritis] |
| H90 | 796 | Conductive and sensorineural hearing loss |
| C79 | 794 | Secondary malignant neoplasm of other and unspecified sites |
| C78 | 774 | Secondary malignant neoplasm of resp and digestive organs |
| S72 | 766 | Fracture of femur |
| E05 | 748 | Thyrotoxicosis [hyperthyroidism] |
| R35 | 718 | Polyuria |
| R65 | 717 | Symp and signs specifically assoc w sys inflam and infct |
| G20 | 696 | Parkinson's disease |
| K52 | 688 | Other and unsp noninfective gastroenteritis and colitis |
| I38 | 678 | Endocarditis |
| R47 | 671 | Speech disturbances |
| Z43 | 663 | Encounter for attention to artificial openings |
| I26 | 648 | Pulmonary embolism |
| H20 | 640 | Iridocyclitis |
| E04 | 628 | Other nontoxic goiter |
| F34 | 618 | Persistent mood [affective] disorders |
| C77 | 614 | Secondary and unspecified malignant neoplasm of lymph nodes |
| M62 | 614 | Other disorders of muscle |
| R01 | 614 | Cardiac murmurs and other cardiac sounds |
| W10 | 606 | Fall on and from stairs and steps |
| H11 | 598 | Other disorders of conjunctiva |
| E89 | 591 | Postproc endocrine and metabolic comp and disorders |
| Z94 | 587 | Transplanted organ |
| M50 | 585 | Cervical disc disorders |
| D68 | 578 | Other coagulation defects |
| N17 | 577 | Acute kidney failure |
| H16 | 571 | Keratitis |
| F20 | 561 | Schizophrenia |
| F84 | 561 | Pervasive developmental disorders |
| E61 | 556 | Deficiency of other nutrient elements |
| G35 | 545 | Multiple sclerosis |
| R15 | 526 | Fecal incontinence |
| R26 | 510 | Abnormalities of gait and mobility |
| E21 | 505 | Hyperparathyroidism and other disorders of parathyroid gland |
| K42 | 495 | Umbilical hernia |
| S70 | 494 | Superficial injury of hip and thigh |
| Z922 | 487 | Personal history of drug therapy |
| I42 | 485 | Cardiomyopathy |
| K56 | 454 | Paralytic ileus and intestinal obstruction without hernia |
| C66 | 450 | Malignant neoplasm of ureter |
| L73 | 450 | Other follicular disorders |
| A41 | 449 | Other sepsis |
| R61 | 445 | Generalized hyperhidrosis |
| J96 | 442 | Respiratory failure, not elsewhere classified |
| R56 | 433 | Convulsions |
| E83 | 428 | Disorders of mineral metabolism |
| J33 | 422 | Nasal polyp |
| A49 | 417 | Bacterial infection of unspecified site |
| L89 | 414 | Pressure ulcer |
| K76 | 409 | Other diseases of liver |
| F40 | 404 | Phobic anxiety disorders |
| C18 | 398 | Malignant neoplasm of colon |
| G81 | 394 | Hemiplegia and hemiparesis |
| C34 | 388 | Malignant neoplasm of bronchus and lung |
| D69 | 373 | Purpura and other hemorrhagic conditions |
| M94 | 363 | Other disorders of cartilage |
| C91 | 352 | Lymphoid leukemia |
| R59 | 350 | Enlarged lymph nodes |
| N45 | 346 | Orchitis and epididymitis |
| E14 | 345 | Unspecified diabetes mellitus |
| Y59 | 338 | Vaccines and biological substances causing adverse effects in therapeutic use |
| I45 | 336 | Other conduction disorders |
| F19 | 335 | Other psychoactive substance related disorders |
| C43 | 334 | Malignant melanoma of skin |
| N12 | 328 | Tubulo-interstitial nephritis |
| R12 | 325 | Heartburn |
| E86 | 314 | Volume depletion |
| F22 | 314 | Delusional disorders |
| N13 | 312 | Obstructive and reflux uropathy |
| N93 | 312 | Other abnormal uterine and vaginal bleeding |
| F25 | 309 | Schizoaffective disorders |
| J84 | 296 | Other interstitial pulmonary diseases |
| G51 | 294 | Facial nerve disorders |
| F60 | 291 | Specific personality disorders |
| K81 | 282 | Cholecystitis |
| K83 | 281 | Other diseases of biliary tract |
| D63 | 278 | Anemia in chronic diseases classified elsewhere |
| B80 | 274 | Enterobiasis |
| F79 | 273 | Unspecified intellectual disabilities |
| H72 | 273 | Perforation of tympanic membrane |
| M89 | 273 | Other disorders of bone |
| K86 | 272 | Other diseases of pancreas |
| I82 | 263 | Other venous embolism and thrombosis |
| M31 | 261 | Other necrotizing vasculopathies |
| G93 | 259 | Other disorders of brain |
| D86 | 256 | Sarcoidosis |
| I12 | 255 | Hypertensive chronic kidney disease |
| I61 | 237 | Nontraumatic intracerebral hemorrhage |
| M07 | 237 | Enteropathic arthropathies |
| G91 | 235 | Hydrocephalus |
| K85 | 235 | Acute pancreatitis |
| I46 | 232 | Cardiac arrest |
| N35 | 230 | Urethral stricture |
| E07 | 229 | Other disorders of thyroid |
| N03 | 225 | Chronic nephritic syndrome |
| G21 | 224 | Secondary parkinsonism |
| I07 | 224 | Rheumatic tricuspid valve diseases |
| E23 | 221 | Hypofunction and other disorders of the pituitary gland |
| Z910 | 219 | Personal history of allergy, other than to drugs and biological substances |
| C20 | 214 | Malignant neoplasm of rectum |
| Z75 | 214 | Problems related to medical facilities and other health care |
| F900C | 213 | Attention Deficit Disorder (ADD) |
| J47 | 212 | Bronchiectasis |
| C83 | 209 | Non-follicular lymphoma |
| C90 | 207 | Multiple myeloma and malignant plasma cell neoplasms |
| F29 | 207 | Unsp psychosis not due to a substance or known physiol cond |
| Z918 | 206 | Personal history of other specified risk-factors, not elsewhere classified |
| Z924 | 206 | Personal history of major surgery, not elsewhere classified |
| Z49 | 200 | Encounter for care involving renal dialysis |
| L28 | 196 | Lichen simplex chronicus and prurigo |
| C64 | 193 | Malignant neoplasm of kidney |
| I78 | 192 | Diseases of capillaries |
| D75 | 189 | Other and unsp diseases of blood and blood-forming organs |
| F11 | 184 | Opioid related disorders |
| N47 | 183 | Disorders of prepuce |
| R58 | 181 | Hemorrhage |
| E29 | 180 | Testicular dysfunction |
| L23 | 177 | Allergic contact dermatitis |
| C25 | 174 | Malignant neoplasm of pancreas |
| J98 | 171 | Other respiratory disorders |
| L70 | 170 | Acne |
| I27 | 169 | Other pulmonary heart diseases |
| G80 | 165 | Cerebral palsy |
| I89 | 165 | Oth noninfective disorders of lymphatic vessels and nodes |
| L43 | 160 | Lichen planus |
| R18 | 157 | Ascites |
| E06 | 150 | Thyroiditis |
| H27 | 148 | Other disorders of lens |
| M32 | 147 | Systemic lupus erythematosus (SLE) |
| U82 | 143 | Resistance to betalactam antibiotics |

**Table S1.** International Classification of Disease (ICD) codes, tenth (ICF-10) revision, for diagnoses used as potential determinants in this study.
